# A Variational Bayesian Deep Network with Data Self-Screening Layer for Massive Time-Series Data Forecasting

**DOI:** 10.3390/e24030335

**Published:** 2022-02-25

**Authors:** Xue-Bo Jin, Wen-Tao Gong, Jian-Lei Kong, Yu-Ting Bai, Ting-Li Su

**Affiliations:** 1 Artificial Intelligence College, Beijing Technology and Business University, Beijing 100048, China; jinxuebo@btbu.edu.cn (X.-B.J.); gongwentao@st.btbu.edu.cn (W.-T.G.); baiyuting@btbu.edu.cn (Y.-T.B.); sutingli@btbu.edu.cn (T.-L.S.); 2 China Light Industry Key Laboratory of Industrial Internet and Big Data, Beijing Technology and Business University, Beijing 100048, China

**Keywords:** time-series data forecast, data self-screening layer, variational inference, gated recurrent unit, maximal information distance coefficient

## Abstract

Compared with mechanism-based modeling methods, data-driven modeling based on big data has become a popular research field in recent years because of its applicability. However, it is not always better to have more data when building a forecasting model in practical areas. Due to the noise and conflict, redundancy, and inconsistency of big time-series data, the forecasting accuracy may reduce on the contrary. This paper proposes a deep network by selecting and understanding data to improve performance. Firstly, a data self-screening layer (DSSL) with a maximal information distance coefficient (MIDC) is designed to filter input data with high correlation and low redundancy; then, a variational Bayesian gated recurrent unit (VBGRU) is used to improve the anti-noise ability and robustness of the model. Beijing’s air quality and meteorological data are conducted in a verification experiment of 24 h PM2.5 concentration forecasting, proving that the proposed model is superior to other models in accuracy.

## 1. Introduction

With the development of sensors and computer storage technology, people have effectively acquired and stored massive amounts of time-series data. The amount of data has exploded, and data often contain multiple related data variables. For example, to accurately predict the PM2.5 of air pollution, the PM2.5 data of different regions, other air quality data, such as PM10, O_3_, SO_2_, CO, and meteorological factors, such as temperature and humidity, etc., can be collected and used. In general, researchers think it will be more accurate and effective to forecast based on multiple related data variables, which has become one of artificial intelligence’s current hot research directions. Machine learning and deep learning methods have been mainly used in time-series data forecasting. Among them, traditional machine learning such as support vector regression (SVR) [1], the integrated moving average autoregressive (ARIMA) model [2], linear regression [3], the Markov prediction method [4], etc., have been widely used. However, due to the insufficient performance of nonlinear fitting, they cannot yet model the highly complex and nonlinear data.

Due to the ability of deep learning networks, they have been widely used in many artificial intelligence fields such as image recognition [5,6,7], image classification [8,9,10], and time-series data forecasting. Recurrent neural networks (RNNs) [11], long short-term memory networks (LSTM) [12], gated recurrent units (GRUs) [13], etc., have become effective ways to solve time-series data forecasting. Improved models such as CNN-LSTM [14] and ConvLSTM [15] with convolution operations can extract high-level spatiotemporal features. Although big data can make the prediction model obtain more input data, it will lead to a large amount of redundancy, conflicts, and inconsistency. Therefore, big data does not mean good data, and blindly considering using big data cannot achieve high forecasting accuracy. Another problem for forecasting is that the data collected by sensors was inevitably polluted by noise, which will degrade the model’s learning accuracy or even overfit the training. Therefore, it is essential to enhance the anti-noise ability of the model. The time-series models are based on statistical data, and the model parameters can be estimated through some identification methods [16,17,18,19,20], such as recursive algorithms [21,22,23,24,25] and hierarchical algorithms [26,27,28,29,30].

In applications, understanding and selecting data can effectively improve model training performance and reduce computational costs. Many methods have been used to measure data correlation, such as the Granger causality analysis method [31], mutual information method [32], Spearman rank correlation coefficient [33], Pearson correlation coefficient [34], etc. However, these methods cannot analyze the redundancy between data. Input data with high redundancy cannot improve the modeling and prediction performance, but it will cost more model training time and even decrease the prediction performance.

Aiming at the problem that the prediction accuracy of neural networks is reduced due to a large number of redundant, conflicting, inconsistent, and noisy input data, the innovations of proposed deep learning networks include the following:

(1) The prediction network is constructed with the data self-screening layer. A maximal information distance coefficient (MIDC) with Bayesian hyperparameter optimization is designed to mine the correlation and redundancy of input data simultaneously, effectively extracting useful input information for deep learning networks and eliminating redundancy.
(2) The variational inference structure is introduced into the gated recurrent unit (GRU) to achieve a Gaussian distribution for the networks’ weights and biases, which can enhance the anti-noise ability of the network and effectively improve forecasting accuracy and generalization performance.

The rest of this article is organized as follows: Section 2 introduces related research work in this field, Section 3 presents the proposed method and prediction model in detail, Section 4 gives experiments with the analysis of results to verify the proposed method, and Section 5 discusses the conclusions.

The abbreviations used in this article are shown in Table 1.

## 2. Related Work

Because traditional machine learning is challenging to learn and fit big data due to its simple structure, researchers often apply deep learning methods with strong information mining capabilities. For example, Teng Mengfan et al. [35] combined LSTM and CNN with a core size of 1 × 1 to predict PM2.5 based on the data from different locations. Zhao et al. [36] proposed a data-driven model called the long short-term memory-fully connected (LSTM-FC) neural network using historical air quality data, weather data, weather forecast data, and the day of the week to predict PM2.5 pollution at a specific air quality monitoring station within 48 h. Yeo et al. [37] gave a deep learning model that combines CNN and GRU to predict the PM2.5 concentration of 25 sites in Seoul, South Korea. They used all weather and air quality data observed between 2015 and 2017 to train the deep learning model. Ding et al. [38] proposed deep transfer metric learning for kernel regression (DTMLKR) to combine deep learning and transfer learning (TL) to solve the problem of regression prediction. YongShi et al. [39] designed a neural network (CNN) with different kernel sizes as a strategy network to predict stock price trends and stock transactions. Jin et al. [40] aimed to deal with existing methods’ limitations with poor stability and unsatisfactory forecast accuracy to propose an attention-based Bayesian hyperparameter optimization network for accurate short-term load prediction.

Researchers have to design more complex networks in the research mentioned above according to the complexity of big input data. Compared with traditional machine learning methods, deep learning networks can capture time-series data information due to their high fitting capabilities. In contrast, for big data, even with deep learning networks, two following factors still lead to a decrease in the forecasting performance:

(1) The data redundancy, conflict, and inconsistency will reduce the learning effect and forecasting accuracy. Therefore, we cannot blindly use big data as the network’s input data. It is necessary to analyze their relationship and select the correct data to improve model training performances.
(2) The noise and uncertainty introduced in the process of sensor measurement will cause the classical neural network to overfit during the training process, which will reduce the forecasting performance. The deep learning network operation mechanism must be reformed to make it applicable and robust to noise and improve the anti-noise ability of the network.

In recent years, existing studies have gradually begun considering relationships between input data for forecasting time-series data to improve accuracy. Abdourahamane et al. [41] combined wavelet transformation and the Frank Copula function and proposed a mutual-information-based nonlinear rainfall forecast model by evaluating the relationship between rainfall series. Peng et al. [42] presented a primary and secondary fuzzy cognitive map (PS-FCM) model to explore the causal relationship of haze pollution data. Han et al. [43] proposed a long short-term memory network based on correlation graph attention, which nests the correlation attention mechanism in the graph attention mechanism to strengthen spatiotemporal correlation. Jin et al. [44] proposed a distributed predictor that can overcome irrelevant data and sensor noise with causality coefficients (SCC) by selecting high causality measures as input data.

Although the above methods consider the selection between input data, these methods are all based on correlation analysis. It does not eliminate redundant information because it is often included in highly correlated data. Recently, variational inference has gradually been applied to the forecasting of the deep learning network of time-series data to improve the anti-noise ability of the network. For example, Zhou et al. [45] proposed a Bayesian framework of the variational graph cyclic attention neural network for robust traffic prediction. Similarly, a variational Bayesian network predicts solar radiation [46] and energy price [47]. These papers show that the variational Bayesian method can overcome the influence of uncertain data with noise, improving the prediction accuracy.

## 3. Data Self-Screening-Variational Bayesian GRU Model

This article proposes a maximal information distance coefficient considering the correlation and redundancy between multivariate data. Combined with Bayesian hyperparameter optimization, a self-screening layer with a self-learning optimization function based on different input data is constructed, which significantly improves the applicability of the prediction network. At the same time, we also built a Bayesian GRU deep prediction network combined with variational inference, which overcomes the difficulty of fitting noise in traditional deep learning and improves the model’s prediction accuracy.

**Figure 1:**
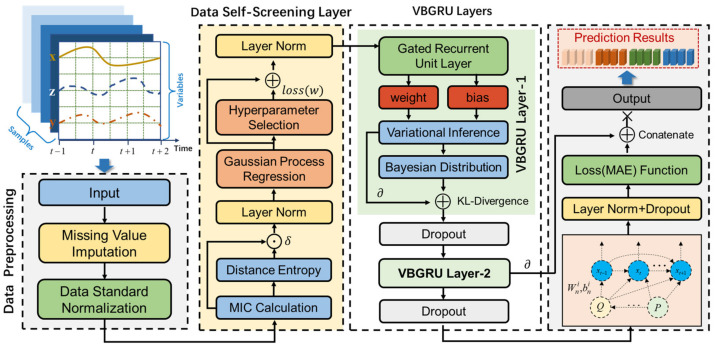
Deep Bayesian prediction network model framework with data self-screening layer.

The framework of the proposed model with the data self-screening layer we designed is shown in Figure 1. The framework mainly includes two parts: data self-screening layer (DSSL) and variational Bayesian gated recurrent unit (VBGRU). The former has maximal information distance coefficient (MIDC) and Bayesian hyperparameter optimization, and the latter mainly combines variational inference and GRU.

The process of this forecasting framework is as the following:

(1) Collect time-series data of multidimensional variables and fill in missing values for the collected data.
(2) Input the processed time-series data into DSSL, which mainly screens variables with high correlation and low redundancy with the target variable and adaptively changes the relevant parameters of the data self-screening layer according to the different input data, then normalize the parameters by layer norm to enhance the suitability of the network.
(3) Input the variables selected by DSSL and target variables into the VBGRU network model for training; then, the dropout layer is used to randomly discard some neural network units to improve the robustness of the model, and finally obtain the prediction results of the target variables.

### 3.1. Data Self-Screening Layer

**Figure 2:**
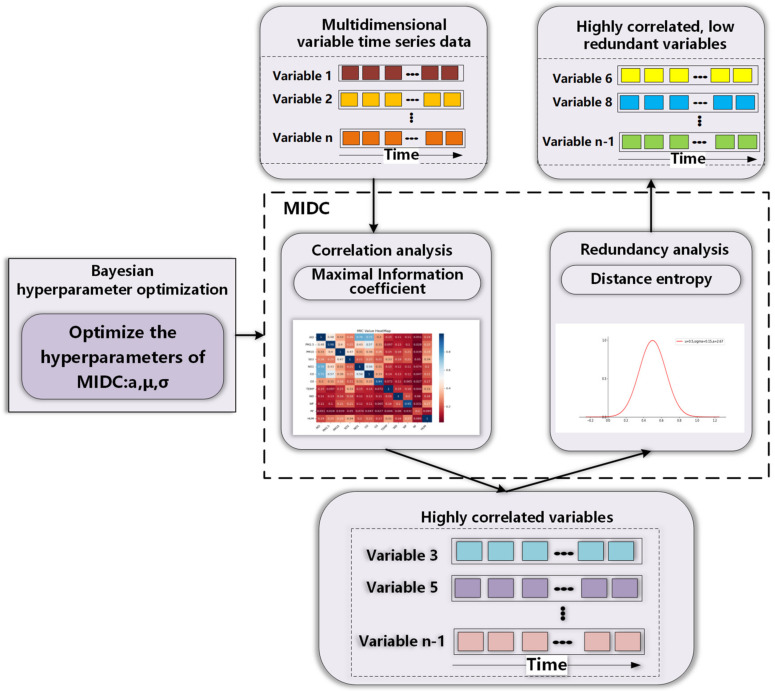
DSSL calculation flow chart.

DSSL comprises two sections: MIDC and Bayesian hyperparameter optimization. MIDC consists of maximal information coefficient (MIC) and distance entropy (DE), and MIC screens the variables with high correlation but low redundancy with the target variable. Bayesian hyperparameter optimization adaptively learns the relevant parameters of MIDC according to the input data. The calculation flow chart of DSSL is shown in Figure 2.

As we know, the maximal information coefficient (MIC) has universality, fairness, and symmetry relative to sequence causality. The calculation of MIC about *x* and *y* is shown in Formula (1):

(1) $$I\left(X;Y\right)=\sum_{x\in X}\sum_{y\in Y}p\left(x,y\right)\log\frac{p\left(x,y\right)}{p\left(x\right)\;p\left(y\right)}$$

**Figure 3:**
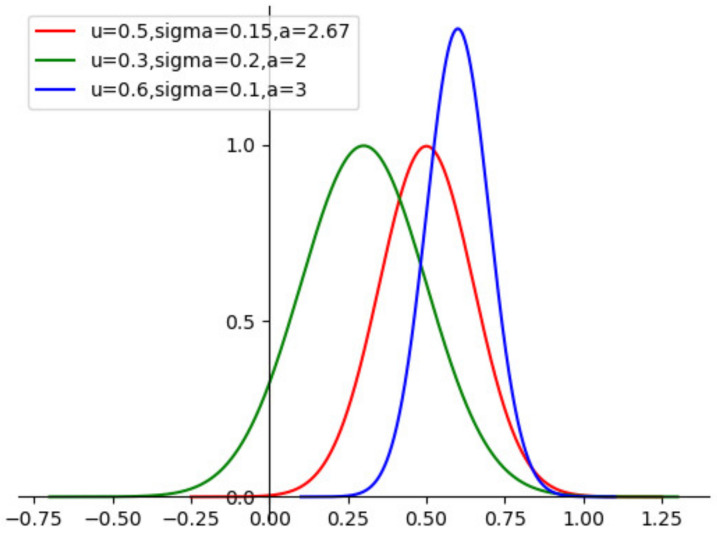
MIDC with different parameters.

However, highly correlated data will contain redundant information in many cases, which is not suitable for neural network training. To select the variables with high correlation but low redundancy, we propose MIDC as the following:

(2) $$\delta \left(I(X;Y)\right)=\frac{\alpha }{\sigma \sqrt{2\pi }}\cdot e^{-\frac{{\left(I(X;Y)-\mu \right)}^{2}}{2\sigma^{2}}}$$

where α,σ,μ are the parameters. The distance coefficient diagram under different parameters is shown in Figure 3. We can see that the different parameters α,σ,μ can convert I(X;Y) into different MIDC values. The Bayesian hyperparameter optimization method will be used to obtain the correct parameters.

The root mean square error is used as the objective function for optimizing hyperparameters:

(3) $$loss(w)=\sqrt{\frac{1}{T}\sum_{t=1}^{T}(y_{t}-{\hat{y}}_{t}{)}^{2}}$$

where w=α,σ,μ is the hyperparameter that MIDC needs to optimize, T is the number of input samples, $y_{t}$ is the actual value, ${\hat{y}}_{t}$ is the predicted value, and t is the time index.

The functional relationship between the hyperparameters and the loss function to be optimized solves the hyperparameter set that minimizes loss(w). The process can be expressed as:

(4) $$w*=\underset{w\in W}{\arg\min}\;loss(w)$$

where w* is the optimal parameter determined by Bayesian hyperparameter optimization, w is a set of input hyperparameters, and W is the parameter space of multidimensional hyperparameters.

Bayesian hyperparameter optimization is divided into two steps: Gaussian process regression (GPR) and hyperparameter selection [48]. The pseudocode of Bayesian optimization is shown in Algorithm 1.
**Algorithm 1: Bayesian Hyperparameter Optimization****Input**:  Initial observation set $D_{n}=\{x_{n},y_{n}\}$  Bounds for the search space χ**Output**: ${\left\{x_{n},y_{n}\right\}}_{n=1}^{t}$**for**n=1,2,…, t **do**  Fit the current data sample $D_{n}$ to get the GPR model $G{\left(w\right)}_{n}$
  Solve the extreme points of the objective function loss(w):$w_{n+1}=\underset{w\in W}{\arg\min}loss(w,G{\left(w\right)}_{n})$
  Obtain new samples $\left(w_{n+1},loss{\left(w\right)}_{n+1}\right)$
  Update data sample $D_{n+1}=\left\{D_{n}\left(w_{n+1},loss{\left(w\right)}_{n+1}\right)\right\}$.  Update data self-screening layer parameters**end for**

### 3.2. Variational Bayesian GRU

**Figure 4:**
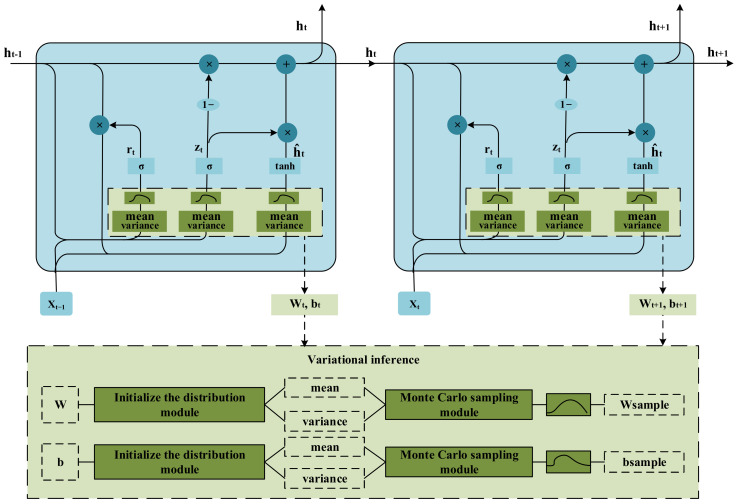
VBGRU structure.

The training process of the deep network model is the process of optimizing the network weights through the sample and target data pairs and finally reaching the desired indicators and obtaining the optimal model weights. Researchers have found that the performance of the deterministic deep network model is affected by the training data itself, resulting in its limited generalization ability. At the same time, the noise in the data source will also affect the network’s performance. For example, when the model is fully trained, it may cause overfitting the noise, resulting in a decrease in prediction accuracy. To solve this problem, researchers have proposed a weight calculation method based on variational inference and applied this method to the training process of deep learning networks to realize the learning of the feature distribution of the original data. The primary theoretical basis of this method is to change the traditional fixed-weight neural network to a distributed-weight neural network. When predicting, the weight distribution is sampled to obtain the prediction result. The network structure of VBGRU is shown in Figure 4.

For deep deterministic networks, the trainable parameters of each network layer correspond to linear or nonlinear changes in input and output, as shown in Formula (5).

(5) $$y^{\left(i+1\right)}=W^{\left(i+1\right)}\cdot y^{\left(i\right)}+b^{\left(i+1\right)}$$

Among them $y^{\left(i\right)}$ represents the activation output corresponding to the i-th layer. In order to realize the flow of information between layers, the weight $W^{\left(i\right)}$ and bias $b^{\left(i\right)}$ of each layer of the trained deep network are composed of specific values. Therefore, a deterministic result will be obtained when using new samples for network testing.

**Figure 5:**
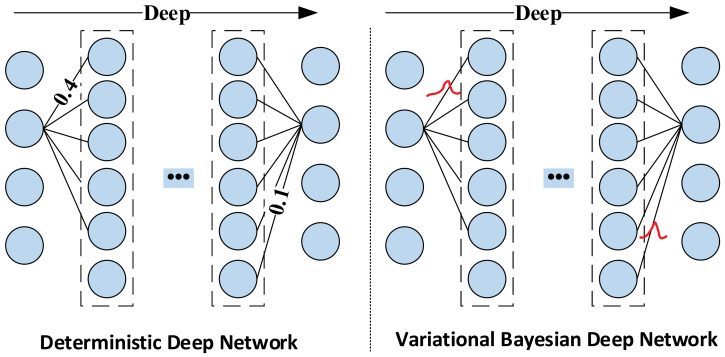
Schematic diagram of the weights of the variational Bayesian network.

Different from the deterministic network, VBGRU selects the weights by sampling from the parameter distribution of the trainable variables calculated in each feedforward calculation, thereby introducing the uncertainty of the weights. As shown in Figure 4, the weights and biases of VBGRU are converted into distribution by the variational inference method. The specific process is: First, initialize the weight W and bias b of the model by initializing the distribution module to obtain the corresponding mean and variance; Then, use the Monte Carlo sampling module to sample the new weight $W_{sample}$ and bias $b_{sample}$ from their corresponding mean and variance. This enables the Bayesian layer to optimize the performance indicators of the model but also learns the uncertainty of network predictions on specific data points. In Figure 5, we show the schematic diagrams of the weights of the deep Bayesian network and the deep deterministic network. VBGRU can obtain multiple model outputs by adding sampling points to calculate the uncertainty of the model at a specific point.

Let $W_{\left(n\right)}^{\left(i\right)}$ denote the n-th sampling weight of the i-th layer and $b_{\left(n\right)}^{\left(i\right)}$ distinguish the bias. In the deep Bayesian network, the weight and bias are not specific numbers but the result obtained by sampling on a distribution. Like the deep deterministic network, the deep Bayesian network is also based on the sample and target data pairs and the weight $W_{\left(n\right)}^{\left(i\right)}$ and bias $b_{\left(n\right)}^{\left(i\right)}$ obtained through training. The difference is that training conveys is not their definite values but their distribution parameters $\rho^{\left(i\right)}$ $\nu^{\left(i\right)}$. The relationship between them and the weight $W_{\left(n\right)}^{\left(i\right)}$ and bias $b_{\left(n\right)}^{\left(i\right)}$ are shown in formulas (6) and (7).

(6) $$W_{\left(n\right)}^{\left(i\right)}=Ν(0,1)*\log(1+\rho^{\left(i\right)})+\nu^{\left(i\right)}$$

(7) $$b_{\left(n\right)}^{\left(i\right)}=Ν(0,1)*\log(1+\rho^{\left(i\right)})+\nu^{\left(i\right)}$$

Generally, a loss function that can be differentiated is required in a deep learning model, and the average absolute error (MAE) is usually used as the loss function. Additionally, the deep Bayesian network’s optimization goal needs to be determined by the loss function. The deep Bayesian network calculates the Kullback–Leibler (KL) divergence between a complex and simple distribution. The loss function is defined as follows:

Let P(ω) be a manually set low-entropy prior distribution,$Q\left(\omega |\theta \right)$ be the posterior distribution of a given parameter. For each scalar weight W obtained for sampling, the KL divergence of the low-entropy prior distribution P(ω) and the posterior distribution $Q\left(\omega |\theta \right)$ follows the formula (8).

(8) $$D_{KL}\left(Q\left(\omega |\theta \right)||P\left(\omega \right)\right)=\underset{n\to \infty }{\lim}1/n\sum_{i=0}^{n}Q\left(\omega^{\left(i\right)}|\theta \right)*\left(\log Q\left(\omega^{\left(i\right)}|\theta \right)-\log P\left(\omega^{\left(i\right)}\right)\right)$$

When n is infinite, we have:

(9) $$D_{KL}\left(Q\left(\omega |\theta \right)||P\left(\omega \right)\right)=1/n\sum_{i=0}^{n}Q\left(\omega^{\left(i\right)}|\theta \right)*\left(\log Q\left(\omega^{\left(i\right)}|\theta \right)-\log P\left(\omega^{\left(i\right)}\right)\right)$$

Since the value of a given parameter $Q\left(\omega |\theta \right)$ can be calculated directly, the KL divergence is calculated by the formula (10).

(10) $$D_{KL}\left(Q\left(\omega |\theta \right)||P\left(\omega \right)\right)=\nu_{Q}*\sum_{i=0}^{n}\left(\log Q\left(\omega^{\left(i\right)}|\theta \right)-\log P\left(\omega^{\left(i\right)}\right)\right)$$

where $\nu_{Q}$ is $\frac{1}{n}\sum_{i=0}^{n}Q\left(\omega^{\left(i\right)}|\theta \right)$, represents the mean value of the posterior distribution, which can be obtained by sampling the posterior distribution multiple times and averaging. Without involving error calculation, it can be excluded from the loss function, and the final loss of each sample can be expressed in the form of Formula (11).

(11) $$Loss=\log\left(Q\left(\omega^{\left(n\right)}|\theta \right)\right)-\log\left(P\left(\omega^{\left(n\right)}\right)\right)$$

In addition, using the KL divergence as the loss error between the prediction result and the network output is not enough because this can only learn the distribution characteristics of the data. Therefore, when training Bayesian GRU, it is usually necessary to combine MAE error to form a connection error, as shown in the formula (12). Among them ∂ represents an error weight parameter, which is generally set as the reciprocal of the number N of all training samples, that is, ∂=1/N.

(12) $$Loss=Loss_{MAE}+\partial \cdot \left[\log\left(Q\left(\omega^{\left(n\right)}|\theta \right)\right)-\log\left(P\left(\omega^{\left(n\right)}\right)\right)\right]$$

Through the above analysis, we initialize all weights and biases in the GRU to a standard normal distribution and update the weight parameters of the network model through the Adam optimizer to obtain the best network parameters. The optimal mean and variance of the weight distribution and bias distribution are obtained. Using the trained model, the weight distribution and bias distribution are sampled multiple times by sampling, and multiple sets of prediction results are obtained. Finally, the multiple sets of prediction results are averaged, which is the predicted value output by the network. Based on the above analysis, the iterative calculation process of the VBGRU model is shown below:

(1) VBGRU initialization: set initialization weight distribution $w_{random}=N\left(0,0.1\right)$, bias distribution $b_{random}=N\left(0,0.01\right)$. Setting parameters $\theta =\left(\rho ,\nu \right)$, The weight $w_{sample}$, and bias $b_{sample}$ after sampling are obtained by Monte Carlo sampling;
(2) Given a total of m samples for each batch: $D\left(X,Y\right)$. Among them X represents the network input data, Y represents the expected output of the network, the network output is $\hat{Y}$;
(3) Use variational inference to sample the network weights and biases n times and calculate the average loss:
  (13) $$Loss=\frac{1}{n}\left\{Loss_{MAE}+\partial \cdot \left[\log\left(Q\left(\omega^{\left(n\right)}|\theta \right)\right)-\log\left(P\left(\omega^{\left(n\right)}\right)\right)\right]\right\}$$
(4) Use Adam optimizer according to Loss to update the weight and bias parameters: $\theta \left(\rho ,\nu \right)$;
(5) Repeat the second to fourth steps of network convergence, that is, Loss no longer drops;
(6) Use the test set to evaluate the trained network model.

## 4. Experiment and Analysis

### 4.1. Data Set Description and Preprocessing

**Figure 6:**
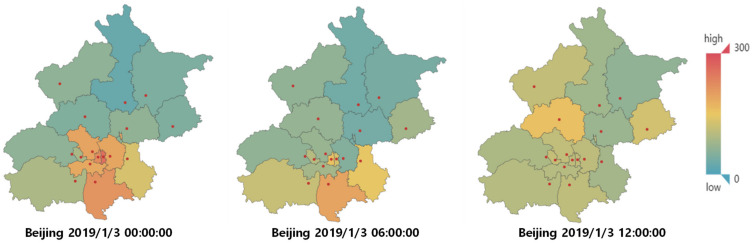
Distribution of PM2.5 in Beijing.

This article uses PM2.5 in the air quality data of Guanyuan in Xicheng District, Beijing, from 1 January 2017, to 31 December 2021, as the target variable. The data sets are the air quality data of neighboring areas and the meteorological data of Haidian, the adjacent area. We performed two types of preprocessing operations: missing value padding and normalization. Each data set contains 43,760 data points, with 90% for the training and 10% for the test. The sampling frequency of all data is 1 h. The air quality data in this data set has a strong temporal and spatial correlation, as shown in Figure 6.

It can be seen that PM2.5 has changed little within six-hour. The reason is that PM2.5 particles have a dissipation process under climatic conditions. In addition, there is a similar process in neighboring areas, and the PM2.5 in heavy industrial development zones is relatively high.

**Figure 7:**
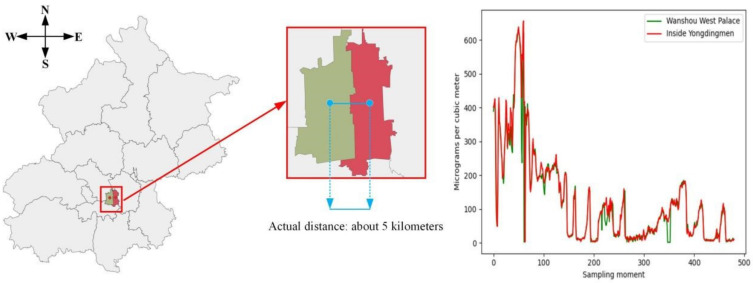
PM2.5 changes in Beijing Wanshou West Palace and Yongdingmen Observatory.

Figure 7 shows the solid spatial correlation in the air quality data. The red area represents Yongding Gate, and the green area represents West Wanshou Palace. It can be seen that these two points are very close. The right figure shows the PM2.5 in these two areas at 480 samplings. The high degree of coincidence proves a redundant relationship between them.

### 4.2. Experiment Establishment and Evaluation Function

Based on our data set, the following two experiments are designed:

(1) DSSL mainly consists of MIDC and Bayesian hyperparameter optimization. MIDC is an essential part of DSSL to analyze and quantify the relationship between different variables. Therefore, the first experiment used MIDC to quantitatively analyze the relationship between the target variable and other air quality factors, select variables with high correlation and low redundancy, and then superimpose these variables in turn and input them into VBGRU to verify the impact of correlation and redundancy between data on prediction performance (see Section 4.3 for details).
(2) Based on the variables filtered out using DSSL, we compare the prediction performance of VBGRU with LSTM, GRU, convolutional long short-term memory network (ConvLSTM), convolutional neural network-long short-term memory network (CNN-LSTM), and time convolutional network (TCN), and evaluate the pros and cons of the model’s predictive ability through the evaluation function (see Section 4.4 for details).

Our experiment used the open-source deep learning library Pytorch to build a deep learning network model. Specifically, our prediction model consists of the DSSL layer, VBGRU layer, and linear layer, and the implicit neuron size of the prediction model is set to 24. The prediction steps are 24. Twenty-four air quality data and weather data on day *i* are used as input objects, and 24 PM2.5 concentration data on day (*i* + 1) are used as expected values. The model is made to perform supervised learning using the Adam optimization algorithm. The learning rate of the Adam optimization algorithm was set to 0.001, and 100 epochs were trained.

Our experiments are conducted on a desktop computer equipped with an AMD R7-5800 processor, 4.0 GHz, and 16GB of RAM. At the same time, we use three evaluation functions to evaluate the prediction performance of the model, namely: root mean square error (RMSE), mean square error (MSE), and mean absolute error (MAE). They are calculated by Formulas (14)–(16), respectively.

(14) $$RMSE=\sqrt{\frac{1}{n}\sum_{i=1}^{n}{\left({\hat{y}}_{i}-y_{i}\right)}^{2}}$$

(15) $$MSE=\frac{1}{n}\sum_{i=1}^{n}{\left({\hat{y}}_{i}-y_{i}\right)}^{2}$$

(16) $$MAE=\frac{1}{n}\sum_{i=1}^{n}|{\left({\hat{y}}_{i}-y_{i}\right)}^{2}|$$

where n represents the total number of samples in the data set, $y_{i}$ representing the actual value of PM2.5, ${\hat{y}}_{i}$ representing the prediction of PM2.5 obtained through experiments. The smaller RMSE, MSE, and MAE, the better the model’s prediction performance.

### 4.3. Performance Verification of MIDC

**Figure 8:**
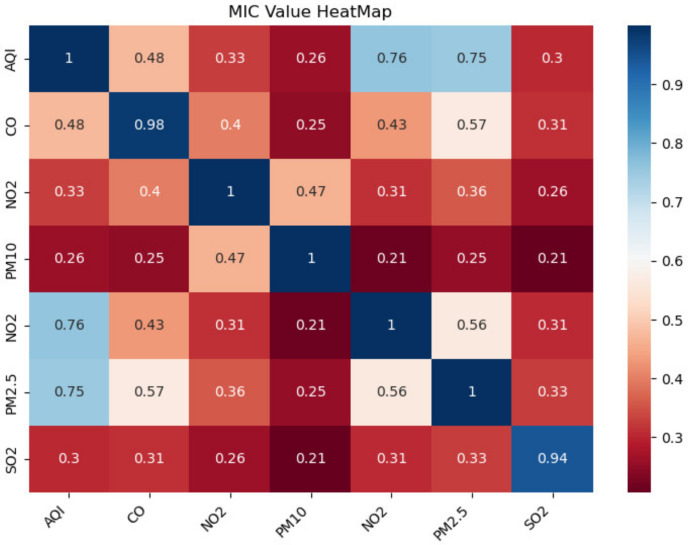
MIC results between different air quality variables in the Guanyuan area.

We first analyzed the correlation between the other six air quality factors, such as AQI, CO, NO_2_, O_3_, PM10, SO_2_, as well as the predicted PM2.5 in the Guanyuan area. The thermodynamic diagram of each variable MIC is shown in Figure 8.

It can be seen that the MICs of PM2.5 and AQI and CO are 0.75 and 0.57, respectively. However, the MIDCs are 0.26 and 0.91, respectively (as shown in Table 2). Table 2 also gives the prediction results of Guanyuan PM2.5 combined with AQI or CO, respectively. It can be seen that when using PM2.5 and AQI as input data, the RMSE is 28.87, and the training time is 48.44 s, while when using PM2.5 and CO as input data, the RMSE is 28.66, and the training time is reduced to 44.81 s.

This result verifies that MIC cannot be used to select input data. The highest MIC indicates high redundancy between AQI and PM2.5, which increases the number of useless computations on the network and reduces the network convergence speed. On the contrary, the proposed MIDC shows a high correlation and can exclude high redundancy.

### 4.4. Compared with Other Models

In this section, we want to compare the performance of the VBGRU model with models such as LSTM [12], GRU [13], CNN-LSTM [14], ConvLSTM [15], and TCN [49] in predicting the hourly PM2.5 concentration in the next 24 h. To better compare the prediction performance of each model, we consider the use of cross-validation methods for performance validation. The commonly used cross-validation methods in machine learning are Monte Carlo simulation [50] and K-fold cross-validation [51]. However, the Monte Carlo simulation method, in which all data samples are randomly sampled after defining the sizes of the training and test machine sets, is not suitable for predicting temporal data with backward and forward dependencies. K-fold cross-validation, also known as rolling cross-validation, is a method that splits the temporal data set into training and test sets according to the temporal order. The results of the K-fold cross-validation runs are averaging can yield an (almost) unbiased estimate of the algorithm performance, avoiding prediction results obtained by chance based on a single iteration for each model, and ensuring accurate comparison of prediction methods.

**Figure 9:**
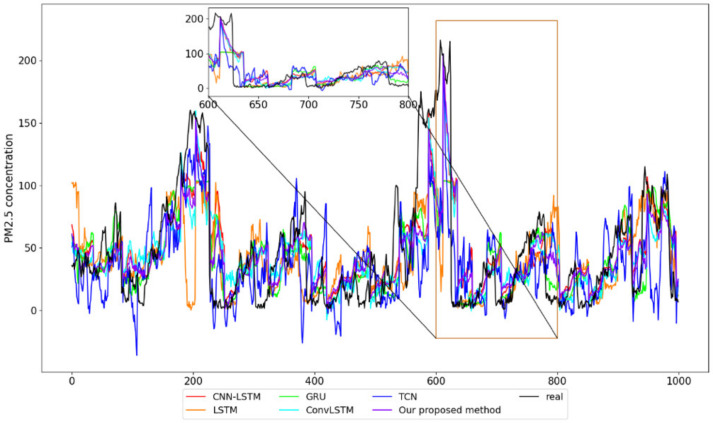
Part of the prediction results of each model.

**Figure 10:**
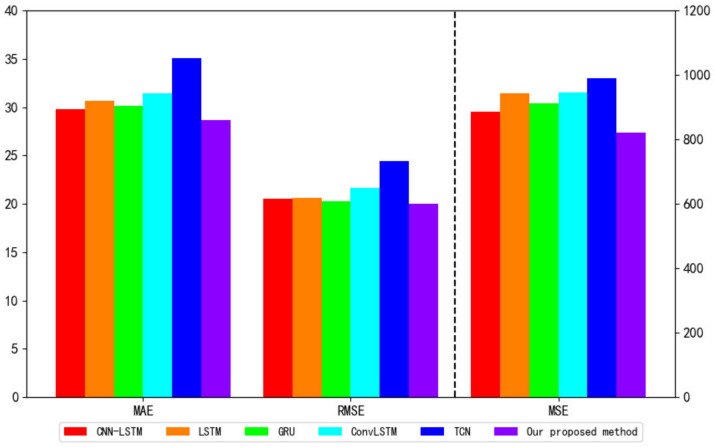
MAE, RMSE, and MSE of different models.

We use 10-fold cross-validation, which divides the dataset into 10 parts in chronological order, and use the first 9 parts of the data in turn as training data in chronological order and the last 1 part of the training dataset as test data to obtain the prediction results of each dataset in turn and carry out the average to obtain the final prediction results. The prediction results of each model are shown in Figure 9 and Figure 10, respectively.

It can be seen from Table 3 that the RMSE, MSE, and MAE of the proposed VBGRU model are 28.59, 817.12, and 19.78, respectively.

Compared with the prediction performance of the other five models, the RMSE of VBGRU decreased by 3.9%, 6.8%, 5.1%, 9.2%, and 18.4% compared to CNN-LSTM, LSTM, GRU, ConvLSTM and TCN, respectively; the MSE decreased by 7.8%, 14.9%, 10.3%, 17.5% and 33.8% respectively; and the MAE of CNN-LSTM, LSTM, GRU, ConvLSTM and TCN decreased by 3.6%, 4.0%, 2.5%, 8.5% and 19.0%, respectively. The method we propose has the smallest prediction error, the best fit to the true value, and the smallest deviation from the true value.

In addition, the training speed of our proposed VBGRU model is 47.71, and the training times of CNN-LSTM, ConvLSTM, and TCN are 69.11, 78.26, and 119.54, respectively, which are much higher than the training speed of the VBGRU model. The training time of GRU and LSTM is 36.73 and 39.97, which is faster than our proposed model, but the prediction accuracy of the two is far lower than our proposed model.

**Figure 11:**
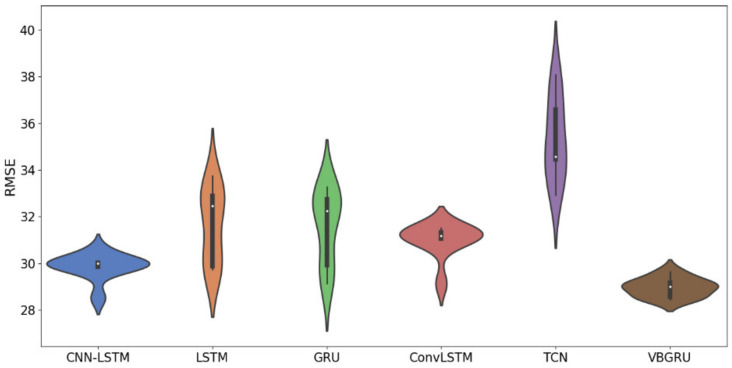
Violin plot of 10 cross-validation results for different models.

To fully demonstrate and compare the prediction performance of each model, we plotted the RMSE of the 10-time cross-validation results of each model into a violin diagram to comprehensively compare the prediction accuracy and robustness of the models. The statistical results are shown in Figure 11.

As shown in Figure 11, our proposed VBGRU has the smallest prediction error range, the most uniform and concentrated distribution, and the smallest average prediction error of the model. It has the highest prediction accuracy and the best stability compared with other models.

Based on the above data analysis, we can conclude that the proposed VBGRU model can achieve better performance at a little computational cost.

## 5. Conclusions and Future Work

Aiming at the problem that a large amount of noise and data conflict, redundancy, or inconsistency reduces the prediction accuracy, this paper proposes a variational Bayesian deep prediction network with a self-screening layer. The model used the self-screening layer to high mine correlation and low redundancy between multiple time-series input variables, reducing unnecessary input information of the model. It gives full play to the powerful feature extraction and anti-noise capabilities of the variational Bayesian GRU for modeling time-series data. It improves the prediction accuracy and robustness effectively.

The prediction and verification experiment of Beijing air quality data, considering the indicators such as RMSE, MSE, and MAE, shows that the model is superior to other models in terms of prediction accuracy and calculation speed. The proposed prediction approaches of time-series models in the paper can combine other parameter estimation algorithms [52,53,54,55,56,57,58] with studying the parameter identification problems of linear and nonlinear systems with different disturbances [59,60,61,62,63,64] to build soft sensor models and prediction models based on time-series data that can be applied to other fields [65,66,67,68,69,70] such as signal processing and engineering application systems [71,72,73,74,75,76,77,78].

## Figures and Tables

**Table 1:** List of abbreviations.

Full Name	Abbreviation
Data self-screening layer	DSSL
Variational Bayesian gated recurrent unit	VBGRU
Maximal information distance coefficient	MIDC
Maximal information coefficient	MIC
Distance entropy	DE
Gaussian process regression	GPR
Kullback–Leibler	KL
Long short-term memory network	LSTM
Gated recurrent unit	GRU
Convolutional long short-term memory network	ConvLSTM
Convolutional neural network-long short-term memory network	CNN-LSTM
Time convolutional network	TCN
Root mean square error	RMSE
Mean square error	MSE
Mean absolute error	MAE

**Table 2:** Forecast results of PM2.5 in the Guanyuan combined with air quality factors.

The Input Data	MIC	MIDC	RMSE	MSE	MAE	Train_Time
PM2.5, AQI	0.76	0.26	28.87	833.48	20.29	48.44 s
PM2.5, CO	0.57	0.91	28.66	821.18	20.02	44.81 s

**Table 3:** Evaluation results of different models on the same data set.

Models	RMSE	MSE	MAE	Train_Time
CNN-LSTM [14]	29.76	886.45	20.51	69.11 s
LSTM [12]	30.66	942.24	20.60	36.73 s
GRU [13]	30.13	911.26	20.28	39.97 s
ConvLSTM [15]	31.45	990.17	21.61	78.26 s
TCN [49]	35.05	1233.42	24.43	119.54 s
Our proposed VBGRU	28.59	817.12	19.78	44.81 s

## Data Availability

Not applicable.
